# Downregulation of TGIF2 is possibly correlated with neuronal apoptosis and autism‐like symptoms in mice

**DOI:** 10.1002/brb3.2610

**Published:** 2022-05-19

**Authors:** Jing Lei, Yijue Deng, Songdong Ma

**Affiliations:** ^1^ Department of the Ninth Pediatrics Hunan Provincial People's Hospital (the First‐Affiliated Hospital of Hunan Normal University) Changsha P. R. China; ^2^ Department of Graduate School Hunan University of Chinese Medicine Changsha P. R. China; ^3^ Hunan Provincial Key Laboratory of Pediatric Respirology Hunan Provincial People's Hospital (the First‐Affiliated Hospital of Hunan Normal University) Changsha P. R. China

**Keywords:** autism, epigenetics, H3K4me1, LSD1, TGIF2, Wnt/β‐catenin

## Abstract

**Background:**

TGFB‐induced factor homeobox 2 (TGIF2) has been reported to exert essential functions in brain development. This study aimed to elucidate the correlation of TGIF2 with autism, a neurodevelopmental condition which presents with severe communication problems.

**Methods:**

An autism‐related gene expression dataset GSE36315 was used to analyze aberrantly expressed genes in autistic brain tissues. Maternal mice were treated with valproate (VPA), and their offspring were selected as model mice with autism. The functions of TGIF2 in autism‐like symptoms in mice were examined by behavioral tests and histological examination of their hippocampal tissues. Mouse hippocampal neurons were extracted for in vitro studies. A gene set enrichment analysis was performed to analyze the signaling pathways involved, and the upstream factors influencing TGIF2 expression were explored in the ENCODE database and validated by ChIP‐qPCR assays.

**Results:**

TGIF2 was poorly expressed in autistic patients in the GSE36315 dataset as well as in the temporal cortex tissues of autistic mice. Adenovirus‐mediated overexpression of TGIF2 suppressed autism‐like symptoms and neuronal apoptosis in autistic mice. TGIF2 activated the Wnt/β‐catenin signaling pathway. TGIF2 could be regulated by monomethylation of histone H3 Lys4 (H3K4me1). The histone demethylase LSD1 was highly expressed in the tissues of autistic mice and bound to TGIF2 promoter, which was possibly responsible for TGIF2 downregulation.

**Conclusion:**

This research suggests that the downregulation of TGIF2, possibly regulated by LSD1/H3K4me1, is correlated with neuronal apoptosis and development of autism in mice through the inactivation of the Wnt/β‐catenin pathway.

## Graphical abstract

This study suggests that TGIF2 is downregulated in autism, which is possibly regulated by LSD1/H3K4me1. Restoration of TGIF2 alleviates neuronal apoptosis and autism‐like symptoms in mouse by activating the Wnt/β‐catenin pathway. The findings suggest that TGIF2 may serve as a candidate tool for the management of autism.

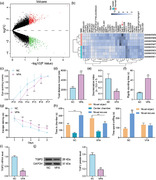

## 1 INTRODUCTION

Since the first description of inborn “autistic disturbances of affective contact” by Leo Kanner, autism has been composed of enigmatic neurobehavioral disorders that frequently become apparent in childhood and then persist as lifelong neuronal impairment (Kanner, 1968; Skinner et al., 2021). The neurodevelopmental condition, also termed autism spectrum disorder (ASD), presents with severe social and communication impairment, restricted interests, and stereotypical and repetitive behaviors (McPartland & Volkmar, 2012; Shuid et al., 2021). Autism is a complex disorder affected by both genetic and environmental factors (Chaste & Leboyer, 2012). The prevalence of autism has seen a substantial increase during the last decade, from 4 to 5 cases per 10,000 children in the 1960s–1970s (Gillberg & Wing, 1999) to approximately 7.6 per 1000 persons in 2010 (Baxter et al., 2015), which might be attributed to upgraded diagnostic standards, increased public awareness, and earlier diagnosis (Shuid et al., 2021).

A recent bio‐collections research by J Reilly et al. documented that hundreds of genes, which are distributed on over 10 chromosomes and are mainly cell adhesion molecules linked to synaptic formation and functions, are correlated with the development of autism (Reilly et al., 2017). In the present study, TGFB‐induced factor homeobox 2 (TGIF2) was identified as one of the downregulated genes in autistic brains via bioinformatics analysis. The TGIFs belong to TALE superfamily of homeodomain proteins (Bertolino et al., 1995). TGIFs were then found to interact with Smads and work as transcriptional co‐repressors that suppress the expression of TGF‐β‐activated genes (Wotton et al., 1999). Of note, the TGIF homeodomain proteins TGIF1 and TGIF2 have been demonstrated to exert essential functions in normal brain development, and their loss was associated with holoprosencephaly (Wotton & Taniguchi, 2018). In addition, TGIF2 has been reported to be highly expressed and play a key role in the mouse retina development (Kuribayashi et al., 2016). Herein, the potential biological involvement of TGIF2 loss in autism development attracted our interests.

During the last decade, inherited or acquired epigenetic changes, which modulate gene expression without altering the DNA sequence itself, have been increasingly recognized to be tightly correlated with stress, health, and diseases (Zhang et al., 2020). Of note, epigenetic factors, including histone modifications, DNA methylation, or noncoding RNA regulations have been documented to play a role in predisposition to autism (Masini et al., 2020). The bioinformatics analysis in this work suggested that there was significant monomethylation of histone H3 Lys4 (H3K4me1) near the TGIF2 promoter. Histone H3K4me1 is one of the enhancer‐specific modifications required for enhancers to activate transcription of target genes (Kang et al., 2021). Importantly, the H3K4me1 has been reported as a marker of the neuronal activity‐dependent enhancers (Malik et al., 2014). LSD1, also known as lysine demethylase 1A (KDM1A), is a histone demethylase which has been reported to suppress H3K4me1 and H3K4me3 modifications and inhibit neurogenesis and impair hippocampal memory in a mouse model of Kabuki syndrome (Zhang et al., 2021). Therefore, the authors surmised that there is an interaction between LSD1, H3K4me1, and TGIF2 in the neural impairment and development of autism. The present study was performed to validate the expression and functions of TGIF2 in autism and the molecular mechanism.

## 2 MATERIALS AND METHODS

### 2.1 Animals

Thirty clean‐level healthy adult C57BL/6J mice, including 15 males (26–28 g) and 15 females (25–27 g), were procured from Hunan SJA Laboratory Animal Co., Ltd (Hunan, China). The animals were housed in 25℃ specific animal rooms with free access to rodent feed and water and given 12 h of light exposure (7:00–19:00) every day. The animal procedures were ratified by the Ethical Committee of Hunan Provincial People's Hospital and abided by the Guide for the Care and Use of Laboratory Animals (NIH Publication No. 85–23, revised 1996).

After 2 weeks of acclimation, 15 pairs of male and female mice were allocated into 15 cages overnight (one male and one female in each cage). On the second day, the vaginal smears from female mice were collected for evaluation. The presence of sperms was considered as an indicator for pregnancy, and the date was recorded as gestational day 0.5. The female mice were then separately housed. The pregnant mice were randomly allocated into two groups, *n* = 8 in one group and *n* = 7 in the other. On gestational day 12.5, the female mice (*n* = 8) were intraperitoneally injected with valproate (VPA; 600 mg/kg, Sigma–Aldrich, Merck KGaA, Darmstadt, Germany; diluted to 250 mg/ml solution with 0.85% normal saline). The newborn mice were allocated into the VPA group. In the other group, the female mice (*n* = 7) were treated with normal saline in a similar manner, and the newborn mice labored by these female mice were allocated into the negative control (NC) group. All female mice were successfully fertilized, and each of them labored 5–12 mice. Mice were raised until young adulthood, and 40 mice were selected from the VPA (*n* = 70) and the NC (*n* = 65) groups for behavioral tests (Rodier et al., 1997). The first day postbirth (P1) was recorded as the date of birth. The eye‐opening time of each mouse was recorded. On day 60, the mice were used to test the development condition and behavioral deficits.

### 2.2 Intracerebroventricular injection

Adenovirus 9 (AAV9)‐based overexpression vector of TGIF2 (AAV9‐TGIF2) and the empty vector (AAV9‐NC) were procured from Genechem Co., Ltd (Shanghai, China). The AAV9‐based vectors were injected into the newborn mice (P1) in the VPA group intracerebroventricularly according to a previous report (Ho et al., 2020). The injection was performed using a small animal stereotactic frame, and the virus was delivered using Hamilton syringes and microinjection adaptors. To prepare the syringe, the borosilicate glass capillary was pulled into a tapered glass needle, and a beveled tip was created by breaking the glass using forceps (∼50–80 μm in diameter). The needle was backfilled using a Luer syringe and 30 G needle, fixed in the Hamilton syringe, and positioned on the frame. AAV9 was loaded into the glass needle through the tip to reduce viral waste. A drop of AAV‐9‐contained phosphate‐buffered saline (PBS) was loaded on the Parafilm beneath the tip, and the syringe plunger was retracted using the frame to draw the virus in a required volume. The glass needles were replaced after every three injections to avoid blunting or clogging. For animal anesthesia, the newborn mice were placed in a chamber infused with 4% isoflurane at a flow of 2 L/min for 3−4.5 min. After 3 min, the anesthesia of mice was examined by a gentle foot pinch at a 30‐s interval. Afterward, the mice were transferred to the anesthetization mould infused with 4% isoflurane at 2 L/min. As for injection, the pipette was placed above the injection site, and the depth axis was zeroed at the height of the skin. The glass needle was rapidly and firmly lowered down to penetrate the skin and skull. Thereafter, the injection depth was adjusted to the desired location to dispense the virus slowly. The injection positions were measured relative to lambda (mm): frontal cortex (Rostral [+]/caudal [−]: +1.5; Lateral: ±1.0, Ventral: 1.0). The injection dose was 0.5 μl and the virus titer was 1 × 10^12^ PFU/ml. The glass pipette was gently retracted from the skull. The tails of injected mice were marked to distinguish them from the uninjected ones. All newborns were placed back to the nest for recovery (10–20 min) as measured by spontaneous limb mobility. Thereafter, the nest was returned to the parent cage.

### 2.3 Behavioral tests

The mice were housed in a constant 24–25℃ condition in a 12‐h light/dark cycle (lights on at 7:00–19:00) with ad libitum access to feed and water. Considering that male autistic children account for most of the reported cases, and young adult/adult mice may behave differently due to the matter of oestrous cycle, only young adult male mice were included for the behavioral tests. At least 3 days before the tests, the mice were housed in the test rooms. Most of the tests were performed as previously reported (Kaminski et al., 2020). The tests were performed during the light‐on hours (9:00–18:00). The interval between every two tests was at least 3 days. The trails of animal movements in all tests were trailed and recorded using an EthoVision 8.0 video tracking system (Noldus Information Technologies, Wageningen, the Netherlands).

### 2.4 Morris water maze tests

The Morris water maze (MWM) tests were performed to examine the learning and memory abilities of mice. The testing system included a movable transparent platform, an automatic recording system, and a round pool. The diameter of the pool was 120 cm, the height was 50 cm, and the depth of water was 30 cm (2 cm over the platform). The temperature of water was adjusted to room temperature. The pool was divided into four quadrants, and the hidden platform was placed in quadrant 1. The mice were acclimated 1 day before the tests, and they were allowed to practice for 3 days. In short, each mouse was placed into the water facing the pool wall in three nonplatform quadrants, respectively. The duration of each test was 60 s. The time for mice spent to find the hidden platform was recorded as the latency. If the mice failed to get to the platform within 60 s, the latency was defined as 60 s. The mean value of the escape latency from three tests was calculated. After 3 days of practice, the latency of each mouse was recorded twice a day for continuous 5 days.

### 2.5 Three‐chamber social test

The three‐chamber social test was performed as previously reported (Silverman et al., 2010) to measure the social behaviors of mice. Each trail lasted for 9 min. One side of the center chamber was an inanimate novel object, and a novel object with a contained mouse was on the other side. Sociability was defined as the subject mice spending more time in the chamber containing the novel target mouse than in the chamber containing the inanimate novel object. Physical contacts around the novel object with nose, head, and forelimbs were deemed as exploration behaviors.

### 2.6 Open field test

The mice were placed in the center of a bright open field arena (75 cm in diameter) as previously described (Kerr et al., 2013). The mice were allowed to explore freely, and the time spent (s) and distance moved (cm) in the inner zone (50 cm in diameter) of the arena as well as the count of entries into the inner zone were analyzed.

### 2.7 Novel object recognition assay

A 40 cm × 40 cm × 40 cm instrument was used for the novel objective recognition assay. On the first day, the mice were allowed to explore freely for 10 min. During the sampling phase on day 2, the male mice were allowed to explore two identical objects for 10 min. During the test phase (24 h later), one of the two objects was replaced with a novel object. The time the mice spent in exploring each of the two objects within a period of 10 min was examined. The cases of mouse nose touching the object or they facing the object within 2 cm were regarded as object recognition. The discrimination index, as a measure of ability to distinguish unfamiliar and familiar objects, was calculated as (*n − f*)/(*n + f*) where “*n*” refers to the time spent in the exploration of the novel object and “*f*” refers to the time spent in exploring the familiar object.

### 2.8 Contextual fear conditioning

On day 1, the male mice were placed in a fear conditioning chamber (Med Associates Inc, VT, USA) and allowed free movement during the first 2 min. Then, a 28‐s tone (85 db) and a 2‐s foot shock (0.75 mA) were applied for three times. After the stimulations, the mice were maintained in the chamber for 30 min. The rigidly standing time of each mouse at each stimulus interval was calculated as a percentage of the freezing response. On day 2, the mice were housed in the same chamber and allowed free movement during the first 2 min to assess environmental memory. On day 3, the chamber was remolded to have the mice placed in a novel condition. The mice were allowed free movement for 2 min and then subjected to 120 s of tone stimulation. The rigidly standing time in environmental trials and tone stimulation trials was recorded, respectively.

### 2.9 Terminal deoxynucleotidyl transferase‐mediated dUTP nick end labeling

An in situ cell death detection kit (Roche Ltd, Basel, Switzerland) was used to examine cell apoptosis in hippocampal tissues. The paraffin‐embedded sections were dewaxed, rehydrated, added with 50 μl proteinase K (20 μg/ml; P6556; Sigma–Aldrich) and hydrolyzed at room temperature for 20 min to remove the tissue protein. After PBS washes and antigen retrieval using citric acid buffer, the sections were reacted with 50 μl TUNEL reaction solution in a wet box avoiding light exposure for 50 min and then washed. The sections were dried, incubated with 50 μl DAPI (Beyotime) at 37℃ in the dark for 30 min sealed with neutral balsam, and observed under the microscope. The apoptotic neurons were observed under the microscope with five random fields selected. The apoptosis index was calculated as apoptotic cells/total cells 100%.

### 2.10 Hematoxylin and eosin staining and Nissl staining

The paraffin‐embedded hippocampal tissue sections were dewaxed, rehydrated, and stained with hematoxylin (Sigma–Aldrich) at room temperature for 5 min. After differentiation in HCl‐ethanol for 30 s, the sections were stained in eosin solution (Sigma–Aldrich) for 2 min. For Nissl staining, the sections were incubated at 37˚C overnight, rehydrated, and stained with Nissl for 10 min. After staining, the tissue sections were routinely dehydrated and sealed for microscope observation.

### 2.11 Reverse transcription‐quantitative polymerase chain reaction

Total RNA from temporal cortex tissues or neurons were extracted using the TRIzol^®^ reagent (Thermo Fisher Scientific Inc., Waltham, MA, USA) and reverse‐transcribed to cDNA using a PrimeScript RT kit (cat. No. RR047A; Takara Holdings Inc., Kyoto, Japan). Thereafter, relative mRNA expression was determined using the 7500 Fast™ System (Applied Biosystems; Thermo Fisher Scientific) and a Sensi Mix SYBR kit (QP100005, OriGene Technologies, Rockville, MD, USA) according to the manufacturer's instruction manual. The primers are presented in Table 1.

**TABLE 1:** Primer sequences for reverse transcription‐quantitative polymerase chain reaction (RT‐qPCR)

Primers	Sequences (5′−3′)
TGIF2	F: CCTCAGAGCAGGAGAAGCTAAG
	R: GGTCTTTGCCATCCTTCCGAAG
LSD1	F: CGATACTGTGCTTGTCCACCGA
	R: CCAAGCCAGAAACACCTGAACC
GAPDH	F: CATCACTGCCACCCAGAAGACTG
	R: ATGCCAGTGAGCTTCCCGTTCAG

Abbreviations: GAPDH, glyceraldehyde‐3‐phosphate dehydrogenase; LSD1, LSD1 zinc finger family protein; TGIF2, TGFB‐induced factor homeobox 2.

### 2.12 Western blot analysis

The tissues and cells were lysed in the radio‐immunoprecipitation assay lysis buffer (Sigma–Aldrich) to collect total protein. The concentration of the isolated protein was examined using the bicinchoninic acid method. Next, an equal amount of protein sample (40 μg each lane) was separated by 10% SDS‐PAGE and loaded onto polyvinylidene difluoride membranes. The membranes were blocked with 5% nonfat milk at 4˚C overnight, and incubated with the primary antibodies against TGIF2 (1:1,000, 11522‐1‐AP; Proteintech Group, Inc., Wuhan, Hubei, China), β‐catenin (1:1,000, #13‐8400; Thermo Fisher Scientific), Wnt1 (1:1,000, #36‐5800; Thermo Fisher Scientific), c‐Myc (1:1,000, ab32072; Abcam Inc., Cambridge, MA, USA), c‐FOS (1:1,000, ab222699, Abcam), Survivin (#PA1‐16836, Thermo Fisher Scientific), and GAPDH (1:10,000; ab8245; Abcam) at 4℃ overnight. Thereafter, the membranes were incubated with HRP‐labeled goat anti‐rabbit IgG (1:5,000; ab6721; Abcam) or goat anti‐mouse IgG (1:2,000; ab6789; Abcam) at room temperature for 1.5 h. The blot bands were developed using the enhanced chemiluminescence system (Thermo Fisher Scientific) and analyzed using Image J software (Version 1.46; NIH, Bethesda, MD, USA).

### 2.13 Extraction and identification of the hippocampal neurons

The hippocampal tissues of the mice in the NC and VPA groups were collected and resuspended in 20% fetal bovine serum at 37℃ using a long glass pipette. The mixture was filtered using a sterile 200‐mesh cytoscreener, centrifuged at 178× *g* for 5 min, and the cells were cultured and resuspended. The cell suspension (2 × 10^6^ cells/ml) was loaded into a sterile culture flask. The medium was replaced by complete medium after 24 h. After 72 h, the medium was added with cytarabine solution (TCI‐C2035; Spectrum Instruments, Shanghai, China) till a final concentration of 2.5 mg/L. The culture solution was refreshed every 3 days. Hippocampal neurons cultured for 6 d were transferred to 96‐well plates at a 1 × 10^4^ cells per well. Afterward, the expression of neuronal markers NeuN and MAP‐2 was examined by immunofluorescence to examine the successful extraction of neurons.

### 2.14 Cell counting kit‐8 (CCK‐8) method

Viability of neurons was examined using the CCK‐8 method (cat. No. CK04; Dojindo Laboratories, Kumamoto, Japan). In short, the cells were seeded in 96‐well plates at 6 × 10^3^ cells per well. Each well was added with 10 μl CCK‐8 solution, followed by 2 h of incubation at 37℃. The optical density at 490 nm was examined using a microplate reader.

### 2.15 Flow cytometry

The hippocampal neurons were cultured in 24‐well plates (1 × 10^5^ cells/well) for 24 h. Annexin V‐fluorescein isothiocyanate (FITC) and propidium iodide (PI; Takara) were added for 10 min of incubation at room temperature in the dark. Apoptosis rate of cells was then examined using a FACS Canto flow cytometer (BD Biosciences, Franklin Lakes, NJ, USA). The Annexin V‐FITC^+^/PI^–^ cells were regarded as apoptotic cells.

### 2.16 Chromatin immunoprecipitation‐qPCR

A Magna ChIP™A/G One‐Day ChIP kit (cat. No. 17–10085; EMD Millipore Corp., Billerica, MA, USA) was used according to the manufacturer's instructions. In short, fresh frozen cortex tissues were cut into 1–3 mm^3^ fragments. The tissue fragments were loaded into 50‐ml tubes, added with 10 ml PBS and then formaldehyde till a final concentration of 1%, rotated at room temperature for 10 min of crosslinking, and then neutralized with glycine for 5 min. The tissues were destructed by SDS lysis buffer (1% SDS, 10 mM EDTA and 50 mM Tris‐HCl; pH 8.0) and treated using a high‐intensity ultrasonic processor on ice at 150 Hz. After centrifugation and collection of the supernatant, an equal amount of chromatin was used for immunoprecipitation at 4℃ overnight. The specific antibodies against LSD1 (ab129195; Abcam) or H3K4me1 (ab176877; Abcam) were used. Anti‐rabbit IgG was used as control, and total chromatin was used as input. After incubation with magnetic beads, the immunoprecipitates were collected. The magnetic beads were washed, and the chromatin was eluted by the proteinase K mixture in ChIP elusion buffer. The TGIF2 expression in the DNA fragments immunoprecipitated by LSD1 or H3K4me1 was examined by qPCR.

### 2.17 Immunofluorescence staining

The hippocampal neurons were fixed with 4% PFA for 15 min, penetrated with 0.5% Triton X 100 at room temperature for 20 min, and blocked with 5% normal goat serum for 30 min. For tissue staining, the paraffin‐embedded cortex tissue sections were dewaxed, rehydrated, boiled in 3% citrate sodium at 100℃ for 20 min, and blocked in the QuickBlock™ Blocking Buffer (cat. No. P0260; Beyotime Biotechnology Co., Ltd., Shanghai, China) at room temperature for 1 h. The sections were incubated with anti‐NeuN (1:500; ab104224; Abcam), anti‐MAP2 (ab183830, Abcam), and anti‐β‐catenin (#13‐8400, Thermo Fisher Scientific) at 4℃ overnight and then with FITC‐conjugated goat anti‐rabbit IgG (1:500; cat. No. A0562; Beyotime) or goat anti‐mouse IgG (1:500, cat. No. A0568, Beyotime) at room temperature for 1 h. The nuclei were stained with DAPI (Beyotime) at room temperature for 5 min. The staining was observed under a fluorescence microscope, and the mean signal intensity was examined using the Image‐pro plus 6.0 software (Media Cybernetics Inc., Bethesda MD, USA).

### 2.18 Dual‐luciferase reporter assay

At 24 h before transfection, the 293T cells (CRL‐3216, ATCC, Manassas, VA, USA) were cultured in six‐well plates for 24 h till a 70% cell confluence. The cells were transfected with 1.0 μg reporter vector BAT‐LUX TCF/LEF (#20890, Addgene, Cambridge, MA, USA) and 50 ng pRL‐TK vector (E2241, Promega, Madison, WI, USA) using Lipofectamine 3000 (Thermo Fisher Scientific). BAT‐LUX induces the expression of a firefly luciferase gene via the seven TCF/LEF binding sites. After 24 h, the medium was refreshed, and the cells were treated with AAV9‐NC or AAV9‐TGIF2. After 48 h, the intensity of firefly and renilla fluorescence in cell lysates was examined using the dual‐luciferase reporter assay system (E1910, Promega) according to the manufacturer's instructions.

### 2.19 Data analysis

The GraphPad Prism 7 software (GraphPa, La Jolla, CA, USA) was used for statistical analysis. Measurement data collected from three independent experiments were expressed as the mean ± SD. Differences were analyzed by the Student's *t* test (two groups) or one‐ or two‐way analysis of variance (ANOVA) followed by Tukey's post hoc test. *p* < .05 was considered to show statistical significance.

## 3 RESULTS

### 3.1 TGIF2 is poorly expressed in autism

**FIGURE 1:**
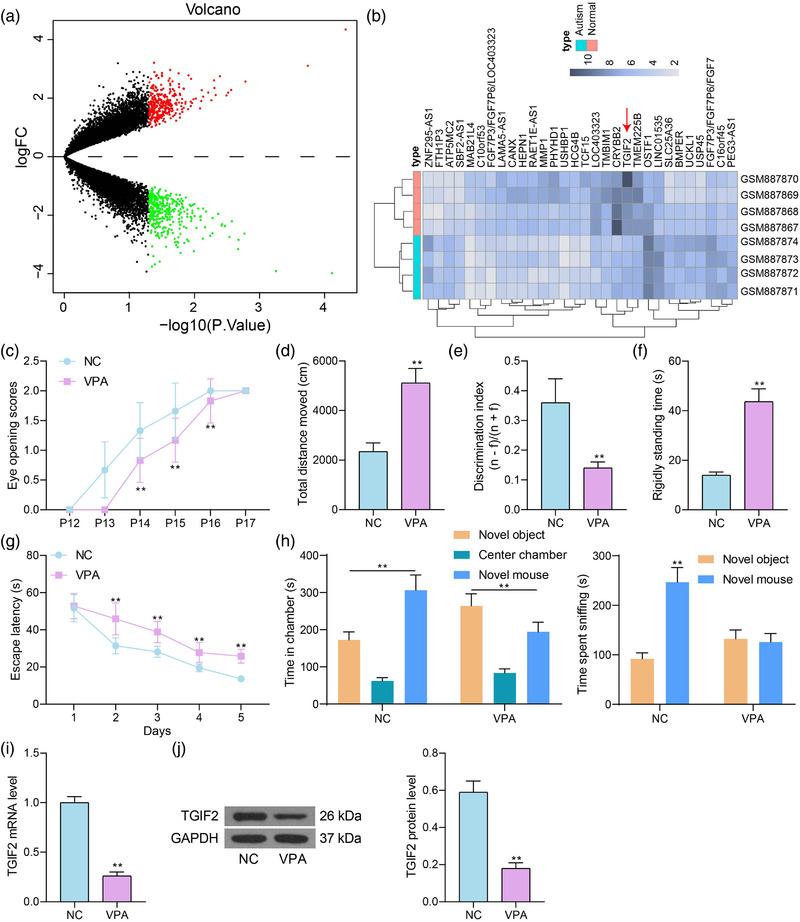
TGFB‐induced factor homeobox 2 (TGIF2) is poorly expressed in autism. (a) A volcano map for the differentially expressed genes (DEGs) between normal brain tissues and autistic brain tissues in the GSE36315 dataset; (b) top 30 DEGs in the GSE36315 dataset; (c) eye‐opening time of each group of mice; (d) anxiety of mice examined by the open field tests; (e) discrimination index of mice examined by the novel object recognition assay; (f) rigidly standing time of mice after stimulations detected by the contextual fear conditioning tests; (g) spatial learning and memory abilities of mice determined by the Morris water maze (MWM) assays; (h) social behavior of mice determined by the three‐chamber social test; (i and j) mRNA (i) and protein (j) levels of TGIF2 in the tissue in temporal cortex of mice detected by reverse transcription‐quantitative polymerase chain reaction (RT‐qPCR) and western blot analysis. In each group, *n* = 6; the behavior tests were observed and recorded by two testers under double‐blind conditions. Data were expressed as the mean ± SD. Differences between groups were analyzed by the unpaired *t* test or two‐way analysis of variance (ANOVA). **p < .01

To identify the possible molecules linked to the pathogenesis of autism, an autism‐related gene expression dataset GSE36315 was downloaded from the GEO database (https://www.ncbi.nlm.nih.gov/geo/) for analysis. The GSE36315 dataset contained four autistic tissue samples and four normal brain tissue samples from healthy individuals. A total of 398 differentially expressed genes (DEGs) were identified (Figure 1a), and the top 30 DEGs are shown in the heatmap in Figure 1b. TGIF2 was identified to be poorly expressed in the brain tissues of autistic patients. Interestingly, the TGIF homeodomain proteins play essential roles in normal brain development (Wotton & Taniguchi, 2018). Therefore, we postulated that the aberrant TGIF2 expression might be correlated with the pathogenesis of autism.

A mouse model with autism was then established. The behavior tests showed that the eye‐opening time of the mice in the VPA group (hereafter termed VPA mice) was significantly delayed compared to the mice in the NC group (hereafter termed NC mice) (Figure 1c). Moreover, the VPA mice had increased anxiety (distance moved), reduced discrimination ability, and increased rigidly standing time after stimulation (Figure 1d–f). Moreover, the MWM tests showed that the escape latency of mice was significantly shortened as the practice time increased. However, the reduction in escape latency was not that significant in VPA mice compared to the NC mice (Figure 1g). Moreover, the three‐chamber social test suggested that the NC mice spent more time in the chamber containing the novel target mouse. By comparison, the VPA mice showed social avoidance behaviors as they spent more time in the chamber containing the inanimate novel object (Figure 1h). These results indicated that the mouse model of autism was successfully established. Thereafter, the expression level of TGIF2 in the brain tissues from temporal cortex of mice was examined. It was observed that the mRNA and protein levels of TGIF2 were significantly reduced in the brain tissues of VPA mice (Figure 1i,j).

### 3.2 Adenovirus‐mediated overexpression of TGIF2 alleviates developmental delay and autism‐like symptoms in VPA mice

**FIGURE 2:**
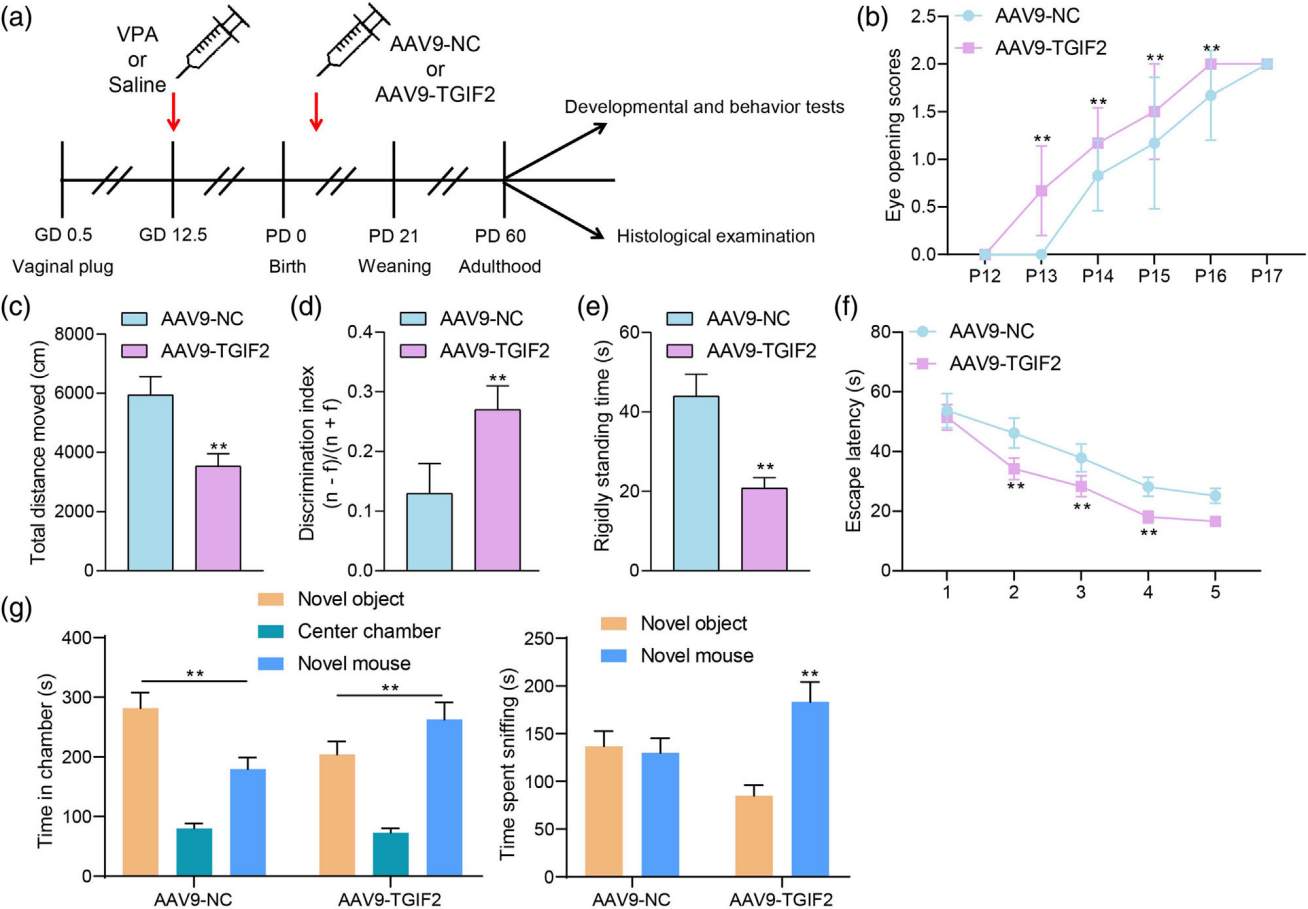
Adenovirus‐mediated overexpression of TGFB‐induced factor homeobox 2 (TGIF2) alleviates developmental delay and autism‐like symptoms in VPA mice. (a) A diagram for the treatment of VPA and injection of AAV9‐TGIF2 or AAV‐NC in mice; (b) eye‐opening time of each group of mice; (c) anxiety of mice examined by the open field tests; (d) discrimination index of mice examined by the novel object recognition assay; (e) rigidly standing time after stimulation of mice detected by the contextual fear conditioning tests; (f) spatial learning and memory abilities of mice determined by the Morris water maze (MWM) assays; (g) social behavior of mice determined by the three‐chamber social test. In each group, *n* = 6; the behavior tests were observed and recorded by two testers under double‐blind conditions. Data were expressed as the mean ± SD. Differences between groups were analyzed by the unpaired *t* test of two‐way analysis of variance (ANOVA). **p < .01

To examine the function of TGIF2 in autistic mice, AAV9‐based overexpression vector containing the TGIF2 cDNA sequence was injected into the VPA mice through intracerebroventricular injection (Figure 2a). After AAV9‐TGIF2 injection, the eye‐opening time of VPA mice shifted to an earlier time (Figure 2b). The distance of mice moved was significantly increased in the open field tests (Figure 2c); the mice showed a marked increase in receptivity to novel things in the novel object recognition assay (Figure 2d); the rigidly standing time of mice after stimulations was significantly reduced in the contextual fear conditioning tests (Figure 2e); and the spatial learning and memory abilities of mice were significantly increased as well, as manifested by reduced escape latency (Figure 2f). Moreover, the three‐chamber social test showed that after AAV9‐TGIF2 treatment, the VPA mice showed reduced social avoidance, as they spent significantly increased time in the chamber containing the novel target mouse (Figure 2g). This body of evidence suggests that TGIF2 overexpression alleviates developmental delay and autism‐like symptoms in VPA mice.

### 3.3 AAV9‐TGIF2 reduces tissue injury in mouse hippocampus

**FIGURE 3:**
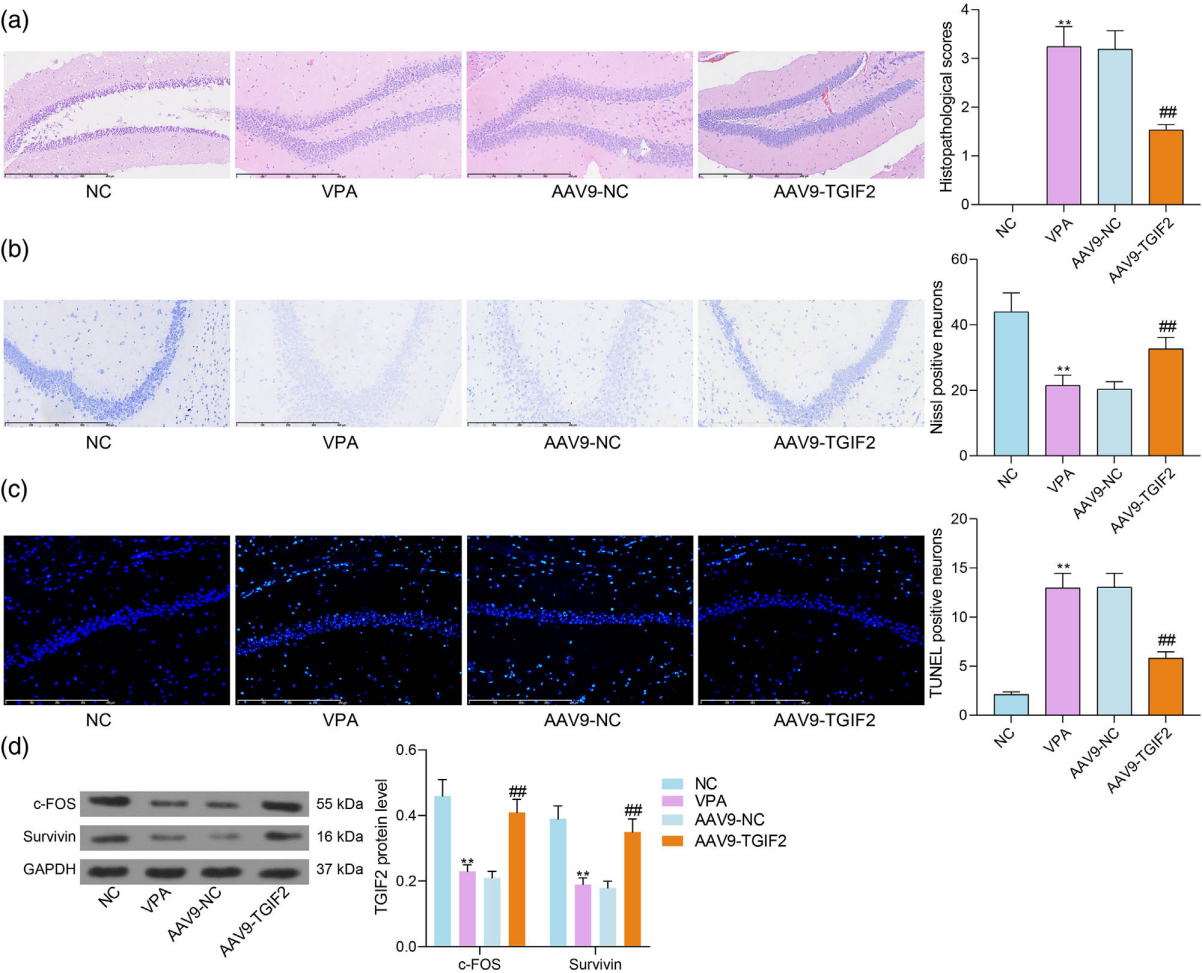
AAV9‐TGFB‐induced factor homeobox 2 (TGIF2) reduces tissue injury in mouse hippocampus. (a) Pathological changes in mouse hippocampal tissues detected by the hematoxylin and eosin (HE) staining; (b) number of active neurons in mouse hippocampal tissues examined by Nissl staining; (c) neuronal apoptosis in mouse hippocampal tissues examined by TUNEL assays; (d) protein levels of neuronal activity‐related c‐Fos and Survivin in mouse hippocampal tissues detected by western blot analysis. In each group, *n* = 6. Data were expressed as the mean ± SD. Differences between groups were analyzed by the one‐way analysis of variance (ANOVA). **p < .01 vs. NC group; ^##^ *p* < .01 vs. the AAV9‐NC group

The pathological changes in mouse hippocampal tissues were detected by the hematoxylin and eosin (HE) staining and Nissl staining. It was observed that AAV9‐mediated TGIF2 overexpression significantly alleviated the neuronal injury in the temporal cortex tissues of VPA mice (Figure 3a). The number of Nissl bodies, which indicated the active neurons, was reduced in VPA mice but restored by AAV‐9‐TGIF2 treatment (Figure 3b). The TUNEL assay suggested that the number of TUNEL‐positive cells (apoptotic cells) was significantly increased in VPA mice but reduced after AAV9‐TGIF2 treatment (Figure 3c). Moreover, the western blot analysis suggested that the levels of neuronal activity‐related proteins c‐Fos and Survivin in hippocampal tissues of the VPA mice were rescued after TGIF2 overexpression (Figure 3d).

### 3.4 Overexpression of TGIF2 increases activity of mouse hippocampal neurons in vitro

**FIGURE 4:**
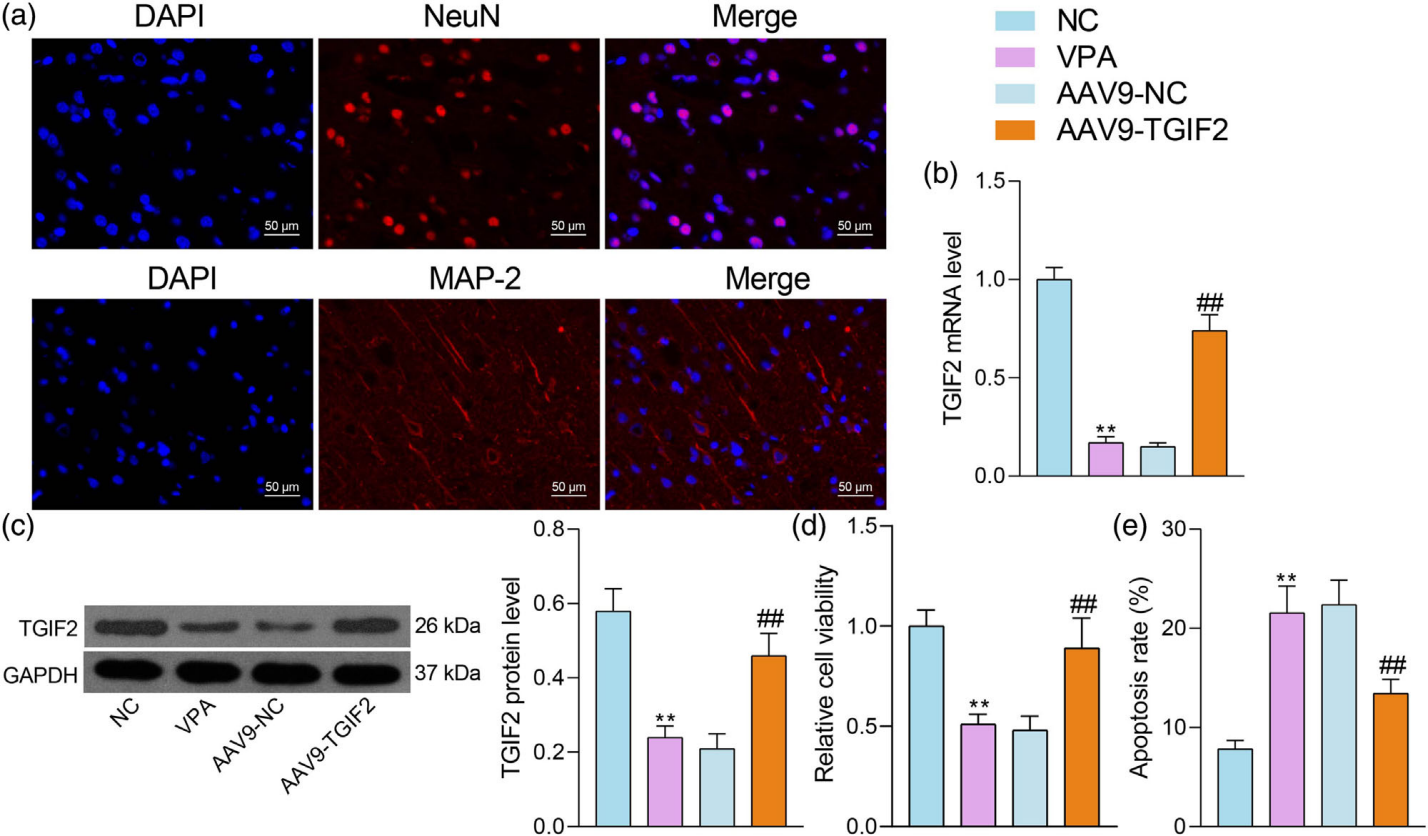
Overexpression of TGFB‐induced factor homeobox 2 (TGIF2) increases activity of mouse hippocampal neurons in vitro. (a) Expression of neuronal markers MAP2 and NeuN in the extracted hippocampal neurons detected by immunofluorescence staining; (b and c), mRNA (b) and protein (c) levels of TGIF2 in hippocampal tissues determined by reverse transcription‐quantitative polymerase chain reaction (RT‐qPCR) and western blot analysis; (d) viability of mouse hippocampal neurons determined by the CCK‐8 assay; (e) apoptosis of cells examined by flow cytometry. Three repetitions were performed. Data were expressed as the mean ± SD. Differences between groups were analyzed by the one‐way or two‐way analysis of variance (ANOVA). **p < .01 vs NC group; ^##^ *p* < .01 vs. the AAV9‐NC group

The hippocampal neurons from NC and VPA mice were extracted and identified by the positive immunofluorescence staining of neuronal markers MAP2 and NeuN (Figure 4a). Moreover, AAV9‐mediated overexpression of TGIF2 was administrated into the hippocampal neurons of VPA mice. Successful upregulation of TGIF2 was detected by reverse transcription‐quantitative polymerase chain reaction (RT‐qPCR) and western blot analysis (Figure 4b,c). Thereafter, the neuronal viability was examined by the CCK‐8 method. It was observed that the viability of neurons of VPA mice was significantly weaker than those of NC mice (Figure 4d). Moreover, the flow cytometry results indicated that the in vitro apoptosis rate of neurons of VPA mice was greater than those of NC mice (Figure 4e). Importantly, upregulation of TGIF2 also increased neuronal viability and reduced neuronal apoptosis in vitro (Figure 4d,e).

### 3.5 TGIF2 regulates the Wnt/β‐catenin pathway in autism

**FIGURE 5:**
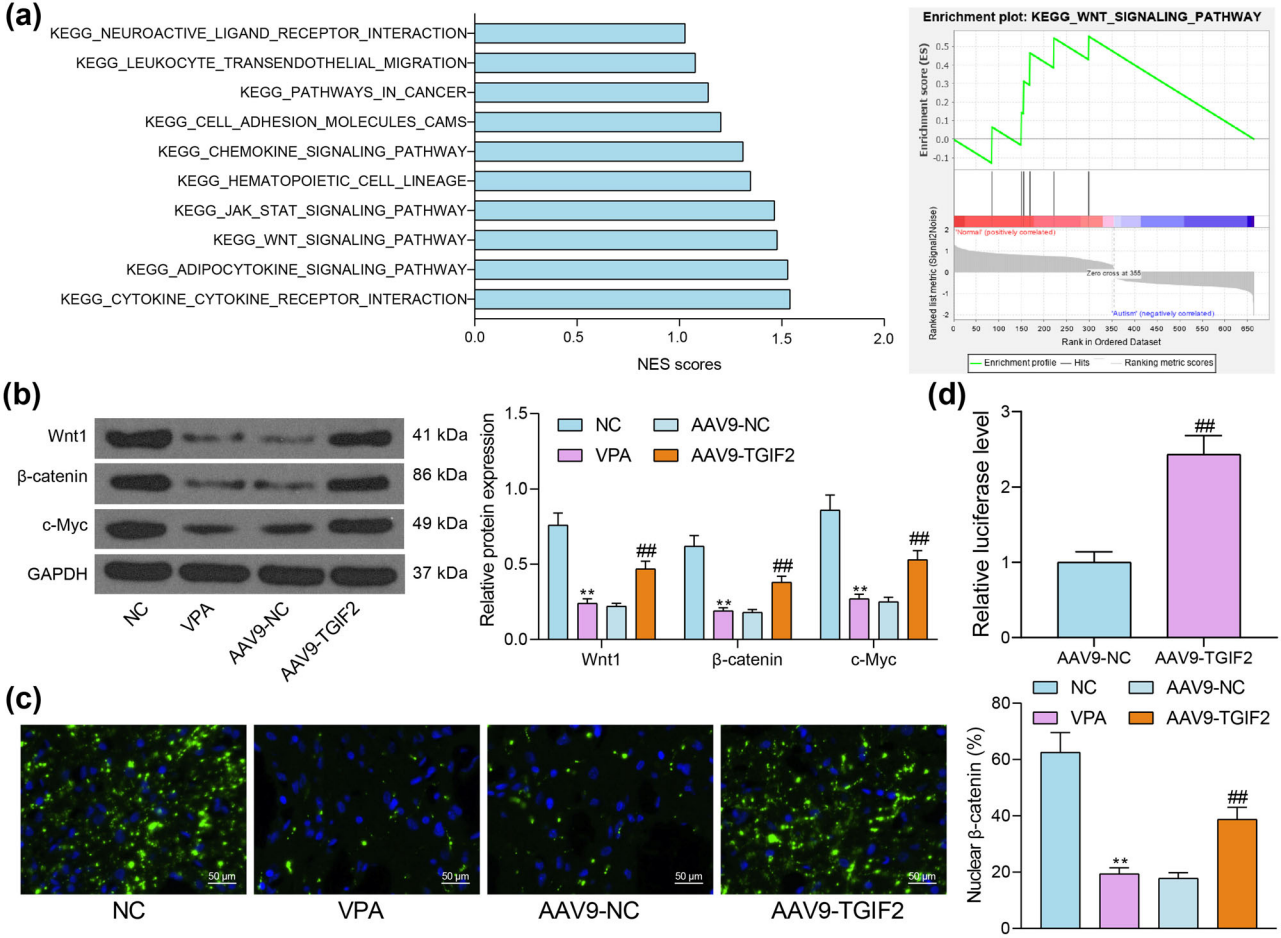
TGFB‐induced factor homeobox 2 (TGIF2) regulates the Wnt/β‐catenin pathway in autism. (a) Signaling pathways enriched by the differentially expressed genes (DEGs) in the GSE36315 dataset analyzed by gene set enrichment analysis (GSEA); (b) levels of the Wnt/β‐catenin pathway‐related proteins Wnt1, β‐catenin, and c‐Myc in the mouse brain tissues examined by western blot analysis; (c) nuclear translocation of β‐catenin in mouse temporal cortex tissues examined by immunofluorescence staining; (d) luciferase activity in 293T cells after TGIF2 overexpression examined by the dual‐luciferase reporter assay. Triplicated wells were set for each experiment. Data were expressed as the mean ± SD. Differences between groups were analyzed by the unpaired *t* test, one‐way analysis of variance (ANOVA), or two‐way ANOVA. **p < .01 vs. NC group; ^##^ *p* < .01 vs. the AAV9‐NC group

To explore the downstream signaling pathways regulated by TGIF2, a gene set enrichment analysis (GSEA) was performed to analyze the signaling pathways enriched by the DEGs in the GSE36315 dataset. It was observed that the Wnt/β‐catenin pathway had the highest correlation with TGIF2 (Figure 5a). Thereafter, the levels of the related proteins were examined by western blot analysis. It was observed that the protein levels of Wnt1, β‐catenin, and c‐Myc in the cortex tissues of VPA mice were significantly reduced but then rescued after TGIF2 overexpression (Figure 5b). In addition, the immunofluorescence staining results showed that the nuclear translocation of β‐catenin in cortex tissues was significantly reduced in VPA mice compared to the NC mice, whereas TGIF2 overexpression promoted the nuclear translocation of β‐catenin (Figure 5c). Moreover, the luciferase assay results indicated that TGIF2 overexpression significantly enhanced the LEF/TCF activity in 293T cells (Figure 5d), indicating that TGIF2 can enhance the activity of the Wnt/β‐catenin signaling pathway.

### 3.6 TGIF2 is regulated by LSD1 and H3K4me1

**FIGURE 6:**
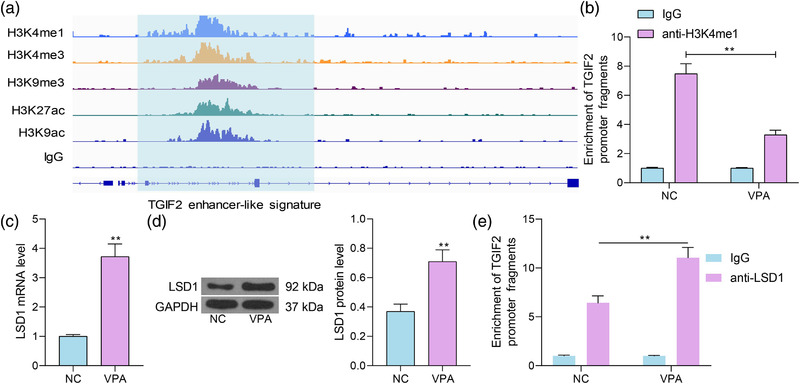
TGFB‐induced factor homeobox 2 (TGIF2) is regulated by LSD1 and H3K4me1. (a) Expression of H3K4me1, H3K4me3, H3K9me3, H3K27ac, and H3K9ac in mouse forebrain tissues in the ChIP‐seq data downloaded from the ENCODE database; (b) H3K4me1 modification level on the TGIF2 promoter in the temporal cortex tissues of VPA mice examined by the ChIP‐qPCR assay; (c and d) mRNA (c) and protein (d) levels of LSD1 in negative control (NC) and VPA mice examined by reverse transcription quantitative polymerase chain reaction (RT‐qPCR) and western blot analysis; (e) binding relationship between LSD1 and the TGIF2 promoter in the mouse temporal cortex tissues examined by the ChIP‐qPCR assay. Three repetitions were performed. Data were expressed as the mean ± SD. Differences between groups were analyzed by the unpaired *t* test, one‐way analysis of variance (ANOVA), or two‐way ANOVA. **p < .01

To examine why TGIF2 was poorly expressed in autism, the ChIP‐seq data including the expression of H3K4me1, H3K4me3, H3K9me3, H3K27ac, and H3K9ac in mouse forebrain tissues were downloaded from the ENCODE database (https://www.encodeproject.org/). It was observed that there was significant H3K4me1 modification near the TGIF2 promoter (Figure 6a). Thereafter, the H3K4me1 level at TGIF2 promoter was examined by the ChIP‐qPCR assay. It was observed that the H3K4me1 modification level on the TGIF2 promoter was significantly reduced in the temporal cortex tissues of VPA mice (Figure 6b). The H3K4me1 has been reported as a marker of the neuronal activity‐dependent enhancers (Malik et al., 2014). Moreover, the histone demethylase LSD1 has been reported to suppress H3K4me1 modification to suppress neurogenesis and impair hippocampal memory in mice (Zhang et al., 2021). Therefore, the expression of LSD1 in the temporal cortex tissues of mice was examined by RT‐qPCR and western blot analysis. It was observed that the expression of LSD1 in VPA mice was significantly increased in the tissues of VPA mice compared to the NC mice (Figure 6c,d). Moreover, LSD1 was able to bind to the TGIF2 promoter through the ChIP‐qPCR assay, and the LSD1 modification level at the TGIF2 promoter was increased in the temporal cortex tissues of VPA mice (Figure 6e). Therefore, downregulation of TGIF2 might be attributed to increased LSD1 level and reduced H3K4me1 modification at its promoter.

## 4 DISCUSSION

Considering the increasing prevalence of autism in children and the accompanying lifelong neuronal disorders (Christensen et al., 2016) while limited treating strategies (Baio et al., 2018), the prevention and treatments of autism are still important health issues. Researchers in this field have made great efforts to identify novel biomarkers for the management of autism (Cheng et al., 2020; Uddin et al., 2017). In this study, the authors report that epigenetic downregulation of TGIF2 is linked to neuronal apoptosis and the neuronal disorder symptoms in mice with autism through the inactivation of the Wnt/β‐catenin pathway.

Integrated bioinformatics analyzing tools including the GEO database have been largely used for the identification of key molecules implicated in the development of diseases, including autism (Li et al., 2014). In this study, TGIF2 was identified as one of the downregulated genes in autistic brains using a GEO GSE36315 dataset. As the temporal and frontal cortices display pathological overgrowth in autistic infants, toddlers, and young children (Carper & Courchesne, 2005; Eyler et al., 2012; Hazlett et al., 2011), and the temporal cortex has been frequently used for the transcriptome analysis in autism (Garbett et al., 2008; Voineagu et al., 2011), the temporal cortex tissues of autistic mice (offspring of VPA‐treated mice) were collected, in which downregulation of TGIF2 was detected. As aforementioned, the TGIF homeodomain proteins have been demonstrated to play essential roles in normal brain development (Wotton & Taniguchi, 2018) as well as in mouse retina development (Kuribayashi et al., 2016). When it comes to the specific role of TGIF2 in autism, the experimental results suggested that AAV9‐mediated TGIF2 significantly alleviated the autism‐like symptoms, such as anxiety, mobility disturbance, impaired learning and memory abilities, and social avoidance. Moreover, the hippocampal neurons were extracted for in vitro experiments due to the convenience in cell extraction and culture. Moreover, the hippocampus is well‐known for its key role in learning and memory formation (Miguez et al., 2015). Loss of hippocampal neurons has been observed in both autistic patients (Meador et al., 2009) and VPA‐induced animal model of autism (Gao et al., 2016). Of note, we found that AAV9‐mediated TGIF2 also suppressed neuronal apoptosis in vitro. This body of evidence suggested that TGIF2 plays a protective role against neuronal apoptosis and neural impairment in autism.

When exploring the downstream signaling pathways using the GSEA, we found that the Wnt/β‐catenin pathway was highly enriched by the DEGs and it had the highest correlation with TGIF2. The levels of Wnt/β‐catenin‐related proteins as well as the nuclear translocation of β‐catenin in the tissues of autistic mice were restored after TGIF2 upregulation. The Wnt/β‐catenin signaling has been widely accepted to be essential for brain development and function and the cascade has a central role in neurodevelopmental pathology in autism (Medina et al., 2018). Indeed, this pathway participates in the processes of neurogenesis (Lie et al., 2005), patterning and maturation of functional synapses (Ciani et al., 2011; Takeichi & Abe, 2005), axonal remodeling (Hollis & Zou, 2012), excitatory neurotransmission (Chen et al., 2006; Sharma et al., 2013), and neuronal differentiation in the developing neocortex (Hirabayashi et al., 2004; Viti et al., 2003). Decreased expression of Wnt and β‐catenin proteins has been observed in the frontal cortex of autistic subjects (Cao et al., 2012). Therefore, TGIF2 possibly activates the Wnt/β‐catenin to restore the neural function in autism.

Like genetic mutations, epigenetic modifications such as histone modifications, DNA methylation, and RNA interference can affect gene expression and possibly lead to behavioral and neuronal changes in mental disorders, including autism (Alam et al., 2017; Masini et al., 2020). Our subsequent bioinformatics analysis suggested that there was a significant H3K4me1 modification at the TGIF2 promoter. H3K4me1 is an active transcription marker and has been suggested as a biomarker of the neuronal activity‐dependent enhancers (Malik et al., 2014). Increased H3K4me1 modification at a newly found enhancer region was associated with transcription during regeneration of injured cortical neurons (Chang et al., 2020). H3K4me1 has been reported to show close correlation with alternative splicing in brain reward tissues (Hu et al., 2017). As aforementioned, LSD1 has been reported to suppress H3K4me1 modification to suppress neurogenesis and impair hippocampal memory in mice (Zhang et al., 2021). LSD1 inhibition showed neuroprotective activity, and one of the major pathways was targeting histone modifications and restoration of the neuroprotective genes and cascades, including the Wnt pathway (Popova et al., 2021). Of note, in the present study, decreased H3K4me1 levels, along with increased LSD1 levels, were detected on the TGIF2 promoter in the temporal cortex tissues of autistic mice. Therefore, it can be inferred that the downregulation of TGIF2 in autism might be attributed to LSD1‐mediated suppression of the H3K4me1 modification at the promoter of TGIF2.

## 5 CONCLUSION

In conclusion, this study demonstrates that TGIF2 is downregulated in autism, which is possibly regulated by LSD1/H3K4me1. Restoration of TGIF2 alleviates neuronal apoptosis and autism‐like symptoms in mouse through activating the Wnt/β‐catenin pathway. The findings suggest that TGIF2 may serve as a candidate tool for the management of autism. We hope more studies will be carried out to validate our findings and offer more insights in the pathogenesis of autism for the development of new therapeutic options.

## CONFLICT OF INTEREST

The authors declare no conflict of interest.

## Data Availability

All the data generated or analyzed during this study are included in this published article.
